# Early Postnatal Treatment with Valproate Induces *Gad1* Promoter Remodeling in the Brain and Reduces Apnea Episodes in *Mecp2*-Null Mice

**DOI:** 10.3390/ijms20205177

**Published:** 2019-10-18

**Authors:** Misa Ishiyama, Satoko Tamura, Hisanori Ito, Hiroki Takei, Manami Hoshi, Masatake Asano, Masayuki Itoh, Tetsuo Shirakawa

**Affiliations:** 1 Department of Pediatric Dentistry, Nihon University School of Dentistry, Chiyoda-ku, Tokyo 101-8310, Japan; ishiyama.misa@nihon-u.ac.jp (M.I.); satocochan@gmail.com (S.T.); itou.hisanori@nihon-u.ac.jp (H.I.); takei.hiroki@nihon-u.ac.jp (H.T.); dema19026@g.nihon-u.ac.jp (M.H.); 2 Department of Pathology, Nihon University School of Dentistry, Chiyoda-ku, Tokyo 101-8310, Japan; asano.masatake@nihon-u.ac.jp; 3 Department of Mental Retardation and Birth Defect Research, National Institute of Neuroscience, National Center of Neurology and Psychiatry, Kodaira, Tokyo 187-8551, Japan; itoh@ncnp.go.jp

**Keywords:** methyl-CpG-binding protein 2 (MeCP2), γ-aminobutyric acid (GABA), glutamate decarboxylase (GAD), valproate, rostral ventrolateral medulla (RVLM)

## Abstract

The deletion of *Mecp2*, the gene encoding methyl-CpG-binding protein 2, causes severe breathing defects and developmental anomalies in mammals. In *Mecp2*-null mice, impaired GABAergic neurotransmission is demonstrated at the early stage of life. GABAergic dysfunction in neurons in the rostral ventrolateral medulla (RVLM) is considered as a primary cause of breathing abnormality in *Mecp2*-null mice, but its molecular mechanism is unclear. Here, we report that mRNA expression levels of *Gad1*, which encodes glutamate decarboxylase 67 (GAD67), in the RVLM of *Mecp2*-null (*Mecp2^-/y^*, B6.129P2(C)-*Mecp2^tm1.1Bird^*/J) mice is closely related to the methylation status of its promoter, and valproate (VPA) can upregulate transcription from *Gad1* through epigenetic mechanisms. The administration of VPA (300 mg/kg/day) together with L-carnitine (30 mg/kg/day) from day 8 to day 14 after birth increased *Gad1* mRNA expression in the RVLM and reduced apnea counts in *Mecp2^-/y^* mice on postnatal day 15. Cytosine methylation levels in the *Gad1* promoter were higher in the RVLM of *Mecp2^-/y^* mice compared to wild-type mice born to C57BL/6J females, while VPA treatment decreased the methylation levels in *Mecp2^-/y^* mice. Chromatin immunoprecipitation assay revealed that the VPA treatment reduced the binding of methyl-CpG binding domain protein 1 (MBD1) to the *Gad1* promoter in *Mecp2^-/y^* mice. These results suggest that VPA improves breathing of *Mecp2^-/y^* mice by reducing the *Gad1* promoter methylation, which potentially leads to the enhancement of GABAergic neurotransmission in the RVLM.

## 1. Introduction

Mutations in the gene encoding methyl-CpG-binding protein 2 (MeCP2) cause Rett syndrome (RTT), a severe neurodevelopmental disorder that affects mostly girls [1,2,3]. RTT patients exhibit autistic symptoms, seizures, stereotypical hand movements, ataxia, and erratic breathing, including breath-holding and life-threatening apneas [4,5,6,7]. Male *Mecp2*-null mice also exhibit respiratory and neurological symptoms, such as an abnormal breathing pattern, hind limb clasping, and gait abnormalities [8,9]. The abnormal breathing is characterized primarily by increased variability in the duration of the respiratory cycle and the occurrence of apneas [10,11,12]. As time advances, breathing frequency decreases, and long-lasting apneas become apparent in *Mecp2*-null mice at 6 weeks and later [10,11].

It has been reported that *Mecp2*-null mice breathe normally at birth and start to show erratic rhythm and overt neurological symptoms in the post-weaning period (3–5 weeks) [8,9,10]. However, subtle but significant differences in behaviors were seen between *Mecp2*-null and wild-type (WT) mice before weaning. The day of the first appearance of postural reflex was postnatal day (PND) 10 in WT male mice while it was PND12 in *Mecp2*-null mice, and *Mecp2*-null mice showed a worse negative geotaxis score than their respective WT littermate controls during the suckling period [13]. Interestingly, reduced inhibitory synaptic inputs to neurons in the rostral ventrolateral medulla (RVLM), a site critical for respiratory rhythmogenesis, were demonstrated as early as PND7 in *Mecp2*-null mice compared to WT controls [14]. These knowledges triggered our interest in the early manifestation of breathing abnormalities and underlying molecular events in *Mecp2*-null mice, which might not be detected with previous approaches.

Valproate (VPA) has been used for decades to treat epilepsy and bipolar mood disorder [15,16]. The increased amount of the inhibitory neurotransmitter γ-aminobutyric acid (GABA) in the brain is one of the proposed effects of VPA treatment, and inhibition of histone deacetylases (HDACs) by VPA is responsible for the mechanism [17]. Epilepsy affects approximately 80% of RTT patients [18], and VPA has been used most commonly for the treatment of epilepsy in RTT [19]. VPA has a rapid onset of action, but a marked increase in anticonvulsant activity is observed during prolonged treatment [16], which supports the hypothesis that epigenetic mechanisms underlie the pharmacological effect of VPA [17,20].

In the present study, mice were treated with VPA or vehicle for 7 days intraperitoneally, and apnea counts were compared between *Mecp2*-null mice developed in the Adrian Bird laboratory (*Mecp2^−/y^* mice) and WT mice on PND15 using whole-body plethysmography. Intervals between each breath were calculated in real-time, and the number of apnea episodes of >1 sec was counted from data recorded for 1 h. The relationship between promoter methylation of *Gad1*, which encodes glutamate decarboxylase 67, and its mRNA expression in the RVLM was examined. We show that *Gad1* mRNA expression in the RVLM was reduced in *Mecp2^-/y^* mice, while VPA increased the mRNA expression and reduced apnea episodes in parallel with the demethylation of the *Gad1* promoter. These findings support a role for VPA in reducing methylation levels of target genes, including *Gad1*, which leads to an improvement of RTT phenotypes represented by erratic control of breathing.

## 2. Results

### 2.1. Spontaneous Apnea Counts are Greater in Mecp2^-/y^ Mice Compared to WT Mice

**Figure 1:**
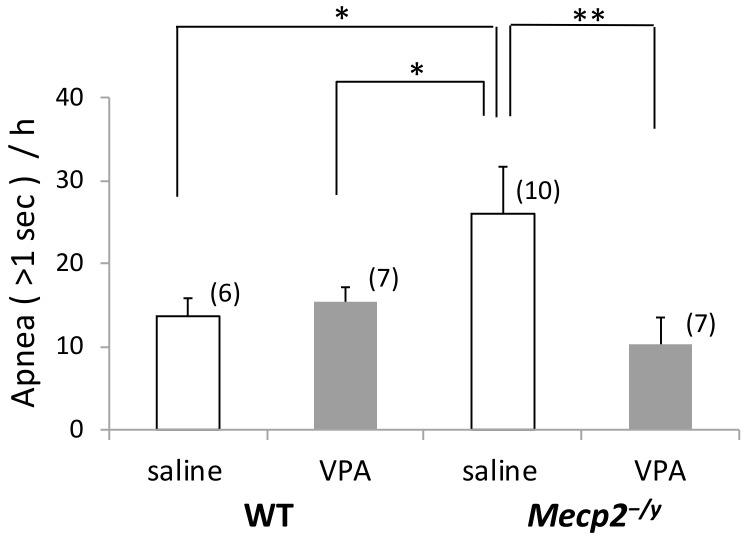
Number of apnea (> 1 s) measured during the 1-h period (10:00–11:00) in 15-day-old mice injected with valproate (VPA) or saline (control) for 7 days. Saline-injected *Mecp2^-/y^* mice displayed an increased number of apneas compared to WT mice, while the number of apnea was reduced in VPA-injected *Mecp2^-/y^* mice. The results of a two-factor ANOVA are as follows; genotype: n.s.; treatment: n.s.; interaction: *p* < 0.05. The asterisks indicate significant differences (* *p* < 0.05, ** *p* < 0.01, Bonferroni test). The numbers of mice belonging to each group are indicated in parentheses. The numbers of mothers that raised mice belonging to each group were 4 (WT-saline), 5 (WT-VPA), 8 (*Mecp2^-/y^*-saline), and 6 (*Mecp2^-/y^*-VPA), respectively. Littermates, if any, belong to the same group.

**Figure A1:**
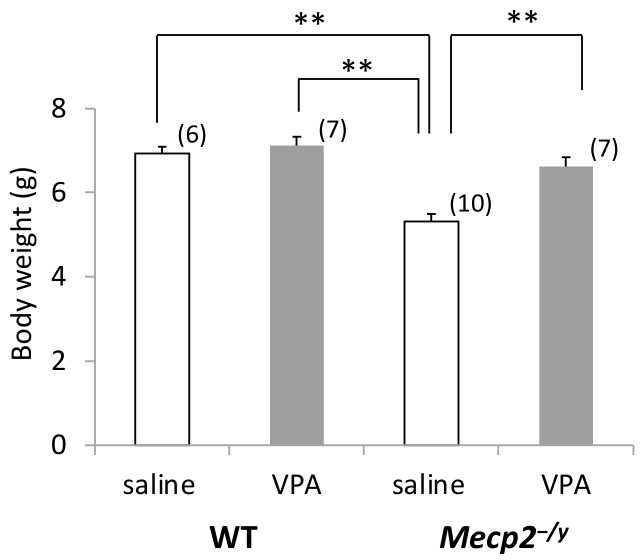
Body weight of mice on PND15. Body weight of saline-injected *Mecp2^-/y^* mice is lower than that of saline-injected WT mice, while VPA treatment significantly increases the body weight of *Mecp2^-/y^* mice. VPA treatment had no effects on the body weight of WT mice. The results of a two-factor ANOVA are as follows; genotype: *p* < 0.01; treatment: *p* < 0.01; interaction: *p* < 0.01. The asterisks indicate a significant difference (** *p* < 0.01, Bonferroni test). The numbers of mice belonging to each group are indicated in parentheses. The numbers of mothers that raised mice belonging to each group were 4 (WT-saline), 5 (WT-VPA), 8 (*Mecp2^-/y^*-saline), and 6 (*Mecp2^-/y^*-VPA), respectively. Littermates, if any, belonged to the same group.

We examined the breathing patterns of mice on PND15 using whole-body plethysmography and found that the *Mecp2^-/y^* mice injected with saline as the vehicle from day 8 to day 14 after birth displayed an increased number of apnea (>1.0 s) episodes compared to the saline-injected WT mice (*p* < 0.05) (Figure 1) although the 15-day old *Mecp2^-/y^* mice did not show long-lasting apneas which emerge during the symptomatic period (about 6 weeks or later after birth) [10,11]. There was no difference in the mean values of respiratory parameters between WT mice and *Mecp2^-/y^* mice (Table A1) except for the number of apneas shown in Figure 1. The mean breathing frequency on PND15 was 235.6 ± 3.4 cycles min^−1^ in *Mecp2^-/y^* mice and 244.6 ± 2.4 cycles min^−1^ in WT mice. The body weight of saline-injected *Mecp2^-/y^* mice was significantly lower than that of saline-injected WT mice on PND15 (Figure A1).

### 2.2. VPA Treatment Reduces Apnea Counts in Mecp2^-/y^ Mice

VPA treatment significantly reduced the number of apneas in *Mecp2^-/y^* mice to the level of WT mice on PND15 (*p* < 0.01) (Figure 1). Other respiratory parameters were also examined in *Mecp2^-/y^* and WT mice that received VPA intraperitoneally from day 8 to day 14 after birth (Table A1). VPA injection did not induce any significant modification of respiratory parameters in *Mecp2^-/y^* mice except for the number of apneas shown in Figure 1. VPA treatment significantly increased the body weight of *Mecp2^-/y^* mice, although it had no effects on the body weight of WT mice (Figure A1).

### 2.3. VPA Treatment Upregulates Gad1 mRNA Expression in the RVLM

**Figure 2:**
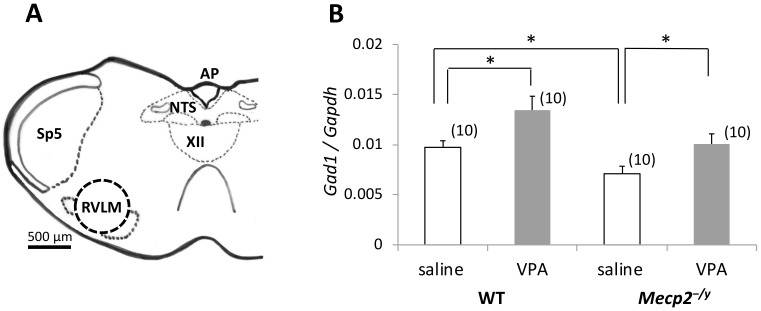
The effects of VPA treatment on *Gad1* mRNA expression in the rostral ventrolateral medulla (RVLM). (**A**) Schematic drawing of a coronal section of the mouse medulla oblongata indicating the position of the caudal end of the RVLM. The border of the punched-out area for RT-qPCR is indicated with a dotted circle. AP: area postrema; NTS: nucleus tractus solitarius; Sp5: spinal trigeminal nucleus; XII: hypoglossal nucleus. (**B**) The graph depicts the levels of normalized *Gad1* mRNA expression in the RVLM of *Mecp2^-/y^* and WT mice. The results of a two-factor ANOVA are as follows; genotype: *p* < 0.01; treatment: *p* < 0.01; interaction: n.s. The asterisks indicate a significant difference (*p < 0.05, Bonferroni test). The numbers of mice belonging to each group are indicated in parentheses. The numbers of mothers that raised mice belonging to each group were 7 (WT-saline), 8 (WT-VPA), 8 (*Mecp2^-/y^*-saline), and 9 (*Mecp2^-/y^*-VPA), respectively. Littermates, if any, belonged to the same group.

Expression of *Gad1* mRNA in the RVLM was examined in *Mecp2^-/y^* and WT mice on PND15 by RT-qPCR (Figure 2) using a primer set (Table A2) designed to target the nucleotide sequence corresponding to the regions in exon18 and exon19 of *Gad1* [21]. *Gad1* mRNA levels in the RVLM of *Mecp2^-/y^* mice injected with saline were lower than that of saline-injected WT mice (*p* < 0.05). However, VPA treatment significantly increased the *Gad1* mRNA level in the RVLM of *Mecp2^-/y^* mice (*p* < 0.05). In addition, VPA treatment also increased the *Gad1* mRNA level in the RVLM of WT mice (*p* < 0.05).

### 2.4. VPA Treatment Enhances Histone Acetylation in the RVLM

**Figure 3:**
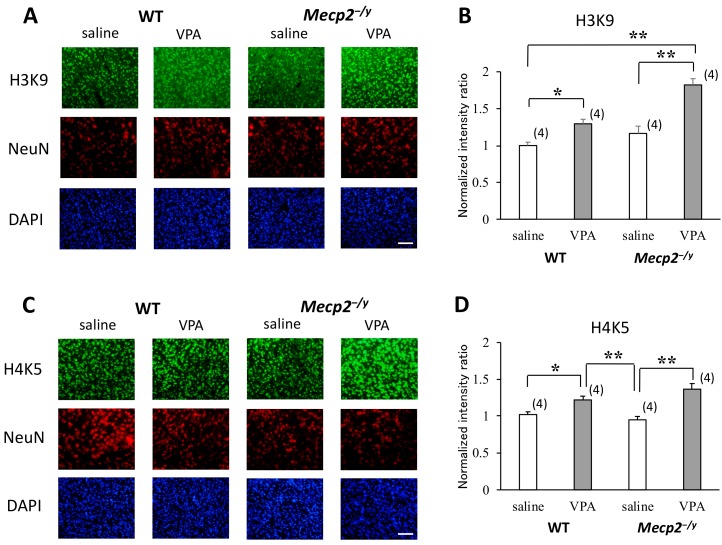
Comparisons of the histone acetylation levels in the RVLM of saline-injected and VPA-injected mice. (**A**,**C**) Representative immunofluorescence staining of cells in the RVLM on coronal sections reacted with antibodies against acetylated lysine residues (Ac-H3K9 and Ac-H4K5) and NeuN. NeuN-immunoreactive cells (red) located in the pre-Bötzinger complex in the RVLM were randomly selected, and the brightness of the nuclei excited with 488 nm (green), which corresponds to each acetylated lysine residue, was analyzed. (**B**,**D**) Normalized ratios of fluorescence intensity calculated using the 8-bit digitized values. VPA treatment increased the acetylation levels of H3K9 and H4K5 in both *Mecp2^-/y^* and WT mice. The results of a two-factor ANOVA are as follows; (**B**) genotype: *p* < 0.01; treatment: *p* < 0.01; interaction: *p* < 0.05; (**D**) genotype: n.s.; treatment: *p* < 0.01; interaction: *p* < 0.05. The asterisks indicate significant differences (* *p* < 0.05, ** *p* < 0.01, Bonferroni test). The numbers of mice belonging to each group are indicated in parentheses. The numbers of mothers that raised mice belonging to each group were 3 (WT-saline), 3 (WT-VPA), 4 (*Mecp2^-/y^*-saline), and 4 (*Mecp2^-/y^*-VPA), respectively. Littermates, if any, belonged to the same group. Scale bar = 50 µm.

**Figure A2:**
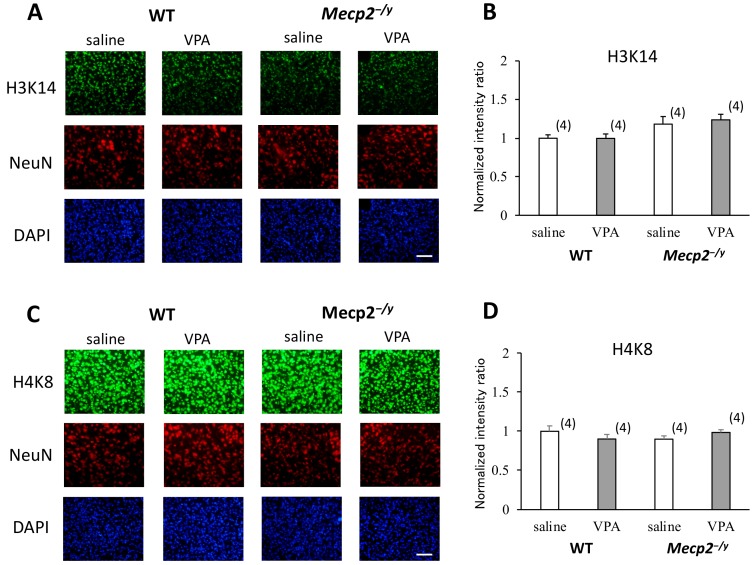
Comparisons of the histone acetylation levels in the RVLM of saline-injected and VPA-injected mice. (**A**,**C**) Representative immunofluorescence staining of cells in the RVLM on coronal sections reacted with antibodies against acetylated lysine residues (Ac-H3K14 and Ac-H4K8) and NeuN. NeuN-immunoreactive cells (red) located in the pre-Bötzinger complex in the RVLM were randomly selected, and the brightness of the nuclei excited with 488 nm (green), which corresponds to each acetylated lysine residue, was analyzed. (**B**,**D**) Normalized ratios of fluorescence intensity. VPA treatment had no effects on the acetylation levels of H3K14 and H4K8 in both *Mecp2^-/y^* and WT mice (n.s., two-factor ANOVA). The numbers of mice belonging to each group are indicated in parentheses. The numbers of mothers that raised mice belonging to each group were 3 (WT-saline), 3 (WT-VPA), 4 (*Mecp2^-/y^*-saline), and 4 (*Mecp2^-/y^*-VPA), respectively. Littermates, if any, belonged to the same group. Scale bar = 50 µm.

For the assessment of acetylation levels of lysine amino acid residues on the tails of histone H3 and H4 in the neurons in the RVLM, we employed an immunofluorescence method using antibodies against acetylated (Ac-) H3K9, Ac-H3K14, Ac-H4K5, and Ac-H4K8. The results showed a significant increase in fluorescence intensity from the nuclei reacted with the Ac-H3K9 or Ac-H4K5 antibody in VPA-injected mice compared to saline-injected mice (Figure 3), while there was not a difference in fluorescence intensity from the nuclei reacted with the Ac-H3K14 or Ac-H4K8 antibody between the saline-injected and VPA-injected groups (Figure A2). Concerning the increased acetylation levels of H3K9 and H4K5, VPA treatment was effective in both *Mecp2^-/y^* and WT mice, and the increase in fluorescence intensity was more prominent in the nuclei of *Mecp2^-/y^* mice compared to WT mice (Figure 3B,D).

### 2.5. VPA Treatment Induces Gad1 Promoter Demethylation in Mecp2^-/Y^ Mice

**Figure 4:**
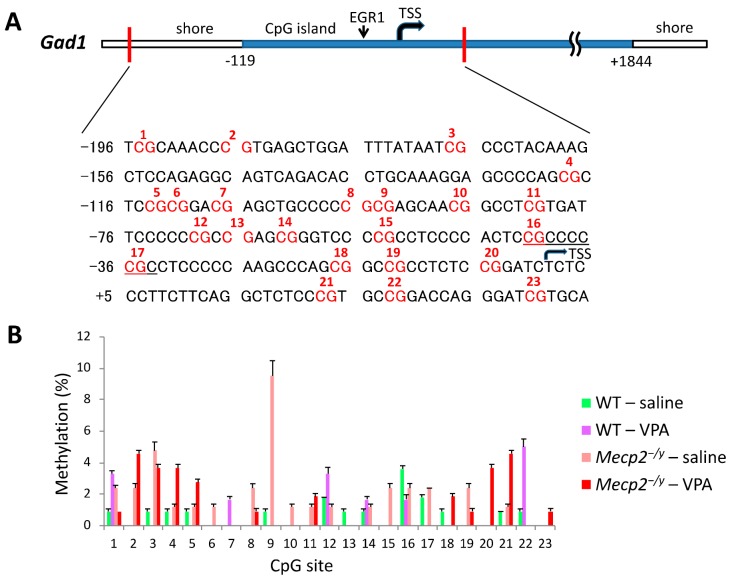
The effects of VPA treatment on the methylation status of individual CpG dinucleotides in *Gad1* promoter. (**A**) A sequence map of the *Gad1* promoter showing 23 CpGs present in the designated region (-196 to +44 with respect to TSS) [24]. The CGCCCCCGC sequence indicates the EGR1-binding motif. (**B**) Mean ± SEM percentage mapping of methylated individual CpGs determined with sodium bisulfite mapping method. Sixty clones were used for the calculation of the percentage of CpG methylation. TSS: transcriptional start site.

**Figure 5:**
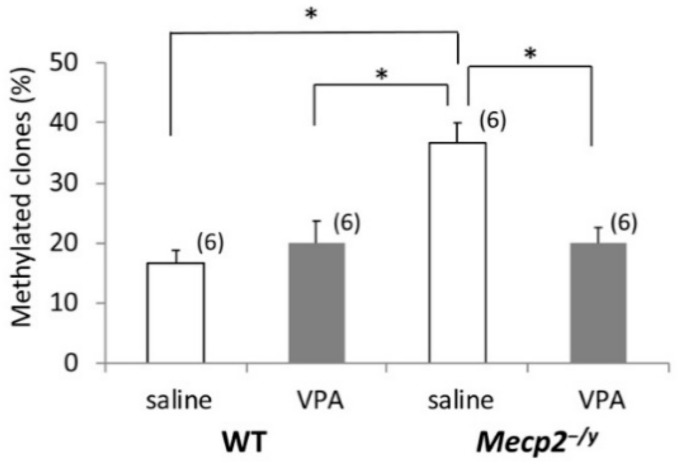
The effects of VPA treatment on *Gad1* promoter methylation. The graph depicts the percentage of methylated clones of the *Gad1* promoter. Clones with one or more methylated sites were classified as “methylated”. The results of a two-factor ANOVA are as follows; genotype: *p* < 0.01; treatment: *p* < 0.05; interaction: *p* < 0.01. The asterisks indicate a significant difference (* *p* < 0.05, Bonferroni test). The numbers of mice belonging to each group are indicated in parentheses. The numbers of mothers that raised mice belonging to each group were 5 (WT-saline), 5 (WT-VPA), 6 (*Mecp2^-/y^*-saline), and 6 (*Mecp2^-/y^*-VPA), respectively. Littermates, if any, belonged to the same group.

We used sodium bisulfite mapping [22] for the determination of DNA methylation status within the *Gad1* proximal promoter (Figure 4A). Cytosine methylation levels in the RVLM extracts were generally low across the targeted *Gad1* promoter region (Figure 4B), as reported previously in the hippocampus of rats [23]. However, we found significant differences in methylation levels between the saline-injected *Mecp2^-/y^* and WT mice, and between the saline-injected and VPA-injected *Mecp2^-/y^* mice (Figure 5). The percentage of methylation in the RVLM was significantly higher in saline-injected *Mecp2^-/y^* mice compared to the mice in three other groups (*p* < 0.05). For the calculation of the percentage of methylated clones in each group, we counted clones with one or more methylated sites as “methylated”, and this value was divided by the total number of clones [23]. The targeted region amplified with our nested PCR primers extends across a shore and a CpG island in the *Gad1* gene, and the transcriptional start site (TSS) exists in this region [24]. In addition, the early growth response protein 1 (EGR1)-binding sequence CGCCCCCGC [23] is included in this region (Figure 4A). When comparisons were made in terms of methylation ratios of individual CpGs, cytosine demethylation occurred mainly within the CpG island upstream to TSS but not in the shore of the *Gad1* promoter in VPA-injected *Mecp2^-/y^* mice (Figure 4B).

### 2.6. VPA Treatment Reduces Tet1 mRNA Expression in Mecp2^-/Y^ Mice

**Figure 6:**
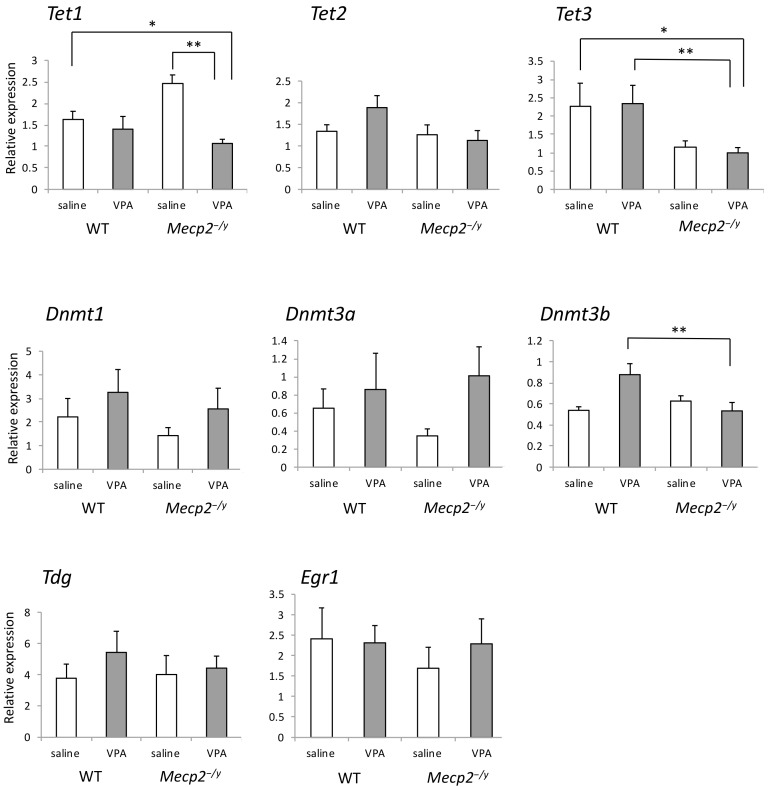
The effects of VPA treatment on the mRNA expression of *Tet1/2/3*, *Dnmt1/3a/3b*, *Tdg*, and *Egr1* in the RVLM determined by RT-qPCR. *Tet1* showed a significantly lower expression level in VPA-injected *Mecp2^-/y^* mice compared to saline-injected *Mecp2^-/y^* mice. In addition, expression levels of *Tet3* and *Dnmt3b* in VPA-injected *Mecp2^-/y^* mice were significantly lower than those in VPA-injected WT mice. The asterisks indicate significant differences (* *p* < 0.05, ** *p* < 0.01, Bonferroni test), and other statistical values are listed in Table A3.

The expression of genes encoding a transcription factor EGR1 and enzymes that can modify DNA methylation was determined using the RVLM extracts by RT-qPCR (Figure 6). Primers used in this procedure are listed in Table A2, and statistical values concerning this experiment are listed in Table A3. Among the eight mRNAs examined, *Tet1* showed a significantly lower expression level in VPA-injected *Mecp2^-/y^* mice (*p* < 0.01) compared to saline-injected *Mecp2^-/y^* mice. Compared with VPA-injected WT mice, expression levels of *Tet3* and *Dnmt3b* were significantly lower in VPA-injected *Mecp2^-/y^* mice. There was no difference in the expression of these mRNAs between *Mecp2^-/y^* and WT mice injected with saline.

### 2.7. VPA Treatment Modifies Mbd1 Association with Gad1 Promoter in Mecp2^-/Y^ Mice

**Figure 7:**
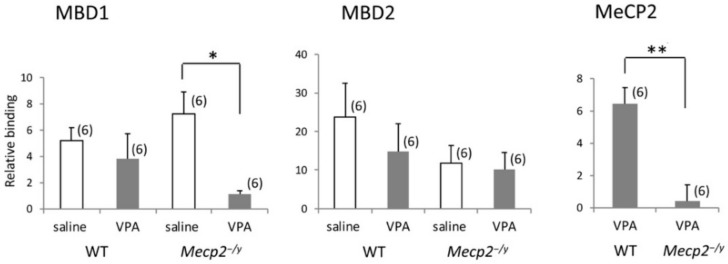
The effects of VPA treatment on the association of MBDs with *Gad1* promoter. VPA downregulated MBD1 binding to the *Gad1* promoter while MBD2 binding was not affected in *Mecp2^-/y^* mice. Neither MBD1 binding nor MBD2 binding to the *Gad1* promoter was affected by VPA in WT mice. Binding of MeCP2 to the *Gad1* promoter was near the detection limit in *Mecp2^-/y^* mice. The results of a two-factor ANOVA are as follows; (MBD1) genotype: n.s.; treatment: n.s.; interaction: *p* < 0.05; (MBD2) genotype: n.s.; treatment: n.s.; interaction: n.s. The asterisks indicate significant differences (* *p* < 0.05, Bonferroni test; ** *p* < 0.01, Student’s *t*-test). The numbers of mothers that raised mice belonging to each group were 5 (WT-saline), 5 (WT-VPA), 6 (*Mecp2^-/y^*-saline), and 6 (*Mecp2^-/y^*-VPA), respectively. Littermates, if any, belonged to the same group.

**Figure A3:**
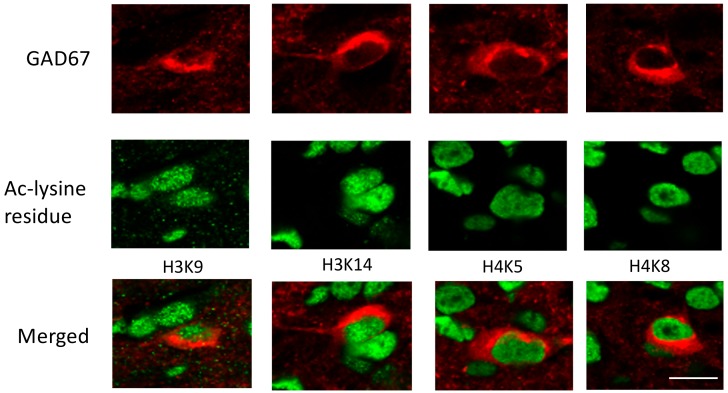
Localization of acetylated lysine residues in GAD67-positive neurons in the neocortex of mice. On PND15, WT mice were deeply anesthetized and perfused transcardially with 4% paraformaldehyde in phosphate-buffered saline. Their brains were rapidly removed and postfixed overnight at 4 °C. Coronal forebrain sections were cut at a thickness of 25 µm. Free-floating sections were incubated with mouse anti-GAD67 monoclonal antibody (1:500; ab26116, Abcam) together with one of the following rabbit polyclonal antibodies, anti-Ac-H3K9 (1:1000); anti-Ac-H3K14 (1:1000); anti-Ac-H4K5 (1:1000); and anti-Ac-H4K8 (1:1000) for 24 h at 4 °C. Then, the sections were incubated for 60 min at room temperature with the appropriate combination of secondary antibodies. The secondary antibodies used were Alexa Fluor 546 goat anti-mouse IgG (1:200; red) and Alexa Fluor 488 goat anti-rabbit IgG (1:200; green). Scale bar = 10 µm.

To investigate the binding properties of MBD1 and MBD2 to the *Gad1* promoter, and the effects of VPA treatment on the DNA-protein interaction, ChIP assays were performed using the mouse forebrain tissues. Tissues from the RVLM were considered more suitable for the purpose, but it was hard to get a sufficient amount of DNA for ChIP from the RVLM of young mice. We confirmed that the glutamate decarboxylase 67 (GAD67)-positive neurons were present abundantly in the neocortex of mice [25], and their nuclei were reactive to antibodies against acetylated lysine residues (Figure A3). The results (Figure 7) showed that VPA downregulated MBD1 binding to the *Gad1* promoter in the brain of *Mecp2^-/y^* mice (*p* < 0.05), although MBD2 binding was not affected. Neither MBD1 binding nor MBD2 binding to the *Gad1* promoter was affected by the VPA treatment in the brain of WT mice. The final qPCR product obtained following ChIP contains 20 CpGs (No.1–20 in Figure 4), and the present results suggest preferential binding of MBD1 to these CpGs in the absence of MeCP2 in *Mecp2^-/y^* mice.

## 3. Discussion

In this study, we demonstrated for the first time that VPA treatment reduced apnea episodes and reduced methylation levels of *Gad1* promoter in 15-day-old *Mecp2*^-/y^ mice. The reduction of apneas occurred in parallel with the reduction of MBD1 binding to the *Gad1* promoter in VPA-injected *Mecp2^-/y^* mice. *Gad1* mRNA expression in the RVLM was upregulated with VPA treatment in both WT and *Mecp2^-/y^* mice. In addition, VPA treatment increased the body weight of *Mecp2^-/y^* mice, presumably through the improvement of GABA-associated control of feeding. *Gad1* encodes GAD67 [26,27], and GAD67 is present mainly in neuronal cell bodies and dendrites and mediates over 90% of basal GABA synthesis [28]. Mice lacking GAD67 showed impairment of the function of the respiratory network [29], and rhythmic activities in the respiratory neural network were not observed in fetal mice lacking the vesicular GABA transporter [30]. Functional roles of GABAergic neurons in phenotypes of RTT were studied in mice with MeCP2 deficiency [31]. In these mice, MeCP2 deficiency in a subset of forebrain GABAergic neurons caused behavioral abnormalities observed in RTT, and the mice exhibited severe respiratory dysrhythmias and premature lethality [31]. MeCP2 expression in the medullary respiratory network is demonstrated to be sufficient for normal respiratory rhythm [32], and the RVLM has been shown to play a crucial role in respiratory rhythmogenesis [12,33]. In *Mecp2*^-/y^ mice with normal resting ventilation, transient apneas with erratic rhythm were occasionally seen at PND15 following hypoxic and hypercapnic challenges [34]. We demonstrate that VPA has the potential to alleviate a mild degree of apneas by reducing methylation levels of *Gad1* promoter and upregulating *Gad1* mRNA expression in the RVLM of pre-weaning *Mecp2*^-/y^ mice. Hence, the dysfunction of the GABAergic system, which causes some symptoms of RTT in *Mecp2*^-/y^ mice at the early stage of life, can be normalized with VPA treatment.

In a recent study, conditional genetic restoration of *Mecp2* expression in GABAergic neurons rescued multiple disease features in a mouse model of RTT [35], which supports the critical regulatory role of GABAergic inhibitory neurons in phenotypes of RTT. GABA is a principal inhibitory neurotransmitter, and GABAergic neurons comprise a functionally confirmed diverse cell population in the mammalian central nervous system [36,37,38,39,40]. It is currently unknown how MeCP2 supports GABA synthesis and how the deletion of *Mecp2* affects pre- and post-synaptic GABAergic inhibitory functions in the brain [14,31,35,37,41,42]. It should be noted that the genetic deletion of a MeCP2-regulated microRNA recapitulated RTT phenotypes in mice bearing MeCP2 intact [43]. There may be a novel mechanism that alters expressions of multiple genes in neurons [44] and induces dysfunction of GABAergic neurotransmission under MeCP2 deficiency. Considering our findings on the methylation status of *Gad1* promoter in *Mecp2*^-/y^ mice, putative target molecules downstream of MeCP2 may play a role in keeping DNA methylation levels at the proper level, and MeCP2 deficiency may interfere with the function.

The acetylation of lysine amino acid residues on the tail of histone H3 and H4 is the most pronounced histone modification and is widely accepted to regulate transcriptional activity [16,17]. VPA has been demonstrated to inhibit HDACs and to induce histone acetylation [45] and DNA demethylation [16,17]. Our study demonstrated that VPA treatment upregulated acetylation of H3K9/H4K5 but not H3K14/H4K8 in the RVLM neurons in both *Mecp2*^-/y^ and WT mice. It is currently unknown how VPA selectively affected the acetylation levels of particular lysine residues. Interestingly, the demethylating effects of VPA on individual CpGs located in the promoter region of the *Gad1* promoter were found to be uneven in our study. As shown in Figure 4B, demethylation occurred preferentially on CpGs positioned in a region in the CpG island -35 to -97 upstream to TSS. Because this promoter region exerts major transcriptional activity as shown in an in vitro experiment [46], our results can be interpreted that demethylation of CpGs in this region caused by VPA treatment reduces binding of the transcriptional repressor MBD1 [47,48,49] and enhances binding of transcription factors, such as EGR1 [23].

It has been demonstrated that DNA methyltransferases (DNMTs) 1, 3a, and 3b are responsible for establishing and maintaining DNA methylation patterns [50] and ten–eleven translocation (TET) family enzymes 1–3 and thymine DNA glycosylase (TDG) are concerned with DNA demethylation [51]. However, in the present experiment, none of the mRNAs encoding these enzymes showed an increase in its expression in response to VPA treatment. Rather, *Tet1* mRNA expression was reduced in VPA-injected *Mecp2*^-/y^ mice, which might have associated with increasing DNA methylation levels and reducing gene expressions in the RVLM. 

Pharmacological therapeutic strategies have been conducted to *Mecp2*-null mice to find out effective drugs against symptoms of RTT [52]. Focusing on a defect in neurotransmitter signaling in the brain, treatments with drugs that target serotonin [53,54], both serotonin and dopamine [55], noradrenaline [10,11], glutamate [56], and GABA [38,39,57] were performed in *Mecp2*-null mice. These trials revealed that the drugs had beneficial effects on the phenotypes and life span of the mice to varying degrees, while few of them succeeded in demonstrating the clinical efficacy. The therapeutic effects of VPA on the symptoms of RTT were evaluated in symptomatic RTT model mice in a recent study [58]. VPA restored the expression of a subset of genes related to neurological functions and alleviated neurological symptoms associated with RTT [58]. Since VPA has been used for decades as a primary drug for the treatment of epilepsy and mood disorder [15,16], our data further support the eligibility of the clinical use of this drug for RTT. Importantly, we have revealed that VPA can normalize a mild degree of erratic breathing observed in pre-weaning *Mecp2*^-/y^ mice. In addition, injections of VPA for 7 days increased the body weight of *Mecp2*^-/y^ mice without obvious side-effects. The present study supports a potential role for VPA in treating or ameliorating symptoms of RTT in early childhood.

## 4. Materials and Methods

### 4.1. Animals

Experiments were performed with approval from the Committee for DNA transformation and the Institutional Animal Care and Use Committee of Nihon University under the approved protocol numbers 2014DEN001 (16 June, 2014) and AP13D026-1 (26 June, 2015). All procedures were reviewed by the Committee in advance of conducting the experiments. Hemizygous *Mecp2^−/y^* mice were generated by crossbreeding heterozygous *Mecp2* mutant females (B6.129P2(C)-*Mecp2^tm1.1Bird^*/J, Jackson Laboratory, Bar Harbor, ME, USA) with C57BL/6J WT males (Sankyo Labo Service, Tokyo, Japan). The mice were provided ad libitum access to food pellets and water at 24 ± 1 °C in a holding room with an alternating 12-h light/dark cycle (lights on at 07:00). Genotyping for pups was performed on postnatal day 7 by PCR following the protocols supplied by The Jackson Laboratory (forward primer: 5′-GGTAAAGACCCATGTGACCC-3′; reverse primer: 5′-TCCACCTAGCCTGCCTGTAC-3′). Male pups born to C57BL/6J wild-type females (WT) were used as control in all the experiments.

### 4.2. VPA Treatment

Mice received intraperitoneal injections of VPA (sodium 2-propylpentanoate, 300 mg/kg body weight, Sigma–Aldrich, St. Louis, MO, USA) and L-carnitine ((3R)-3-hydroxy-4-(trimethylazaniumyl) butanoate, 30 mg/kg body weight, Wako, Tokyo, Japan) dissolved in saline at 18:00 every day from day 8 to day 14 after birth. These mice were nursed together with naïve littermates. Body weight was measured every day before the injection. Control mice received saline as vehicle at the same time each day as the experimental groups. L-carnitine was added to the VPA solution aiming to avoid carnitine deficiency that potentially occurs as a side effect of VPA administration [59]. Given the positive effects of acetyl-L-carnitine, an acetylated derivative of L-carnitine, on some of early RTT symptoms in *Mecp2^1lox^* mice [60], we pretested effects of daily injections of l-carnitine (30 mg/kg body weight) alone from day 8 to day 14 after birth on the respiratory parameters of *Mecp2^−/y^* mice and found no substantial effects.

### 4.3. Plethysmographic Assessment of Apnea Episodes

Breathing waveforms were recorded from unrestrained mice on PND15 by whole-body plethysmography placed in an experimental room maintained at 24 ± 1 °C. WT and Mecp2^−/y^ mice were put in a closed plethysmograph chamber (450 mL volume) connected to a control module (PLY3211, Buxco Electronics, Wilmington, NC, USA) and data were processed and stored in a personal computer (PC) using Biosystem XA software (Buxco Electronics). The chamber was flushed with fresh air at a rate of 1 L/min continuously. Before the recording, mice were placed in the chamber for 30 min intending to habituate them to the environment. Apnea counts, breathing frequency, tidal volume, inspiratory time, expiratory time, peak inspiratory flow, and peak expiratory flow were recorded and analyzed in real-time for 60 min (10:00–11:00).

For the assessment of apnea episodes, we used the flow threshold setting of the software to determine the start time of each breath by detecting the start of inspiration. When the interval between the start time of two consecutive breaths exceeded 1.0 s, we judged the episode as apnea. This criterion is based on our observation in the present study as well as a report from another research group [34] that the mean respiratory cycle of 15-day-old mice is less than 0.3 s. Each apnea episode detected by the software on a breath-by-breath basis was validated by reviewing the breathing waveform on a computer display under playback mode. Only intervals between the start time of two consecutive breaths without body-movement artifacts were used for the analysis.

### 4.4. Isolation of Total RNA and Quantitative Reverse Transcription PCR Analysis

Mice treated with either VPA or saline for 7 days were anesthetized deeply with isoflurane, and whole brains were rapidly removed and frozen on PND15. Using a cryostat (CM1850, Leica, Wetzlar, Germany), frozen brains were mounted on the specimen discs and were cut coronally to expose the caudal end of the RVLM. Bilateral RVLM tissues were removed using an 18-gauge needle according to the mouse brain atlas of Franklin and Paxinos [61] and stored at −80 °C. Total RNA was extracted using the RNeasy mini kit (QIAGEN, Hilden, Germany). The RNA quality and concentration were assayed using a NanoDrop 2000 device (Thermo Fisher Scientific, Waltham, MA, USA). Purified total RNA was reverse-transcribed into cDNA by incubating with a recombinant ribonuclease inhibitor (TaKaRa Bio, Tokyo, Japan), 5× prime script buffer (TaKaRa Bio), 5× reverse transcriptase M-MLV Buffer (TaKaRa Bio), and random primer (Invitrogen, Carlsbad, CA, USA). A quantitative reverse transcription PCR (RT-qPCR) assay was performed with SYBR Premix Ex Taq II (TaKaRa Bio), and data analysis was carried out with the use of CFX96-Real-Time System (Bio-Rad, Hercules, CA, USA). For the quantification of mRNA, the target expression level was normalized to the *Gapdh* (glyceraldehyde-3-phosphate dehydrogenase) mRNA level. The primer sequences used for RT-qPCR are shown in Table A2.

### 4.5. Quantitative Immunofluorescence Labelling

On PND15, mice treated with either VPA or saline for 7 days were deeply anesthetized with sodium pentobarbital (150 mg/kg, intraperitoneal) and perfused transcardially with ice-cold saline followed by 4% paraformaldehyde in phosphate-buffered saline. Their brains were rapidly removed from the skulls. Dissected brains were postfixed overnight at 4 °C in the same fixative, and were cryoprotected by immersion in 30% sucrose and stored at −80 °C.

Coronal sections were cut through the caudal brainstem at a thickness of 25 µm using the CM1850 cryostat (Leica). Free-floating sections were incubated with mouse anti-NeuN monoclonal antibody (1:1000; MAB377, Millipore, Billerica, MA, USA) together with one of the following rabbit polyclonal antibodies, anti-Ac-H3K9 (1:1000; A-4022, Epigentek, Farmingdale, NY, USA); anti-Ac-H3K14 (1:1000; A-4023, Epigentek); anti-Ac-H4K5 (1:1000; A-4027, Epigentek); and anti-Ac-H4K8 (1:1000; A-4028, Epigentek) for 24 h at 4 °C. Then, the sections were incubated for 60 min at room temperature with the appropriate combination of secondary antibodies. The secondary antibodies used were Alexa Fluor 546 goat anti-mouse IgG (1:200; ab60316, Abcam, Cambridge, UK) and Alexa Fluor 488 goat anti-rabbit IgG (1:200; ab150081, Abcam). Preparation and labeling of the brain sections from *Mecp2*^−/y^ mice and WT mice were carried out at the same time, and four pairs of the brains served for this experiment.

The sections were mounted on a glass slide and coverslipped with Fluoroshield mounting medium with 4′,6-diamidino-2-phenylindole (DAPI) (ab104139, Abcam). Immunofluorescent images of bilateral RVLM were digitally captured under constant excitation light using a microscope (Eclipse 80i, Nikon, Tokyo, Japan) equipped with a 20× objective and a CCD camera (DXM1200F, Nikon). On the 8-bit images obtained from the sections, NeuN-positive neuronal nuclei (*N* = 20 per each section) located in the pre-Bötzinger complex were randomly selected, and the brightness of the nuclei excited with 488 nm which corresponds to each acetylated lysine residue was analyzed. DAPI fluorescence intensity was used as a reference.

### 4.6. Sodium Bisulfite Mapping

Cytosine-methylation of *Gad1* promoter was examined using sodium bisulfite conversion followed by DNA sequencing [22]. Mice treated with either VPA or saline for 7 days were anesthetized deeply with isoflurane, and whole brains were rapidly removed on PND15. Then, the brains were frozen, and bilateral RVLM tissues were punched out and stored at −80 °C. Genomic DNA extracted from the RVLM tissues was treated with sodium bisulfite (Imprint DNA Modification Kit, Sigma–Aldrich) for 90 min at 65 °C. To yield enough amount of bisulfite-treated target cDNA, two successive PCRs (nested PCR) were performed on the genomic DNA (GenBank: NC_000068.7) from RVLM tissues. For the first amplification, the following outside primers were used to amplify a 331-bp region corresponding to the *Gad1* proximal promoter, forward: 5′-GTGTTGAGTGTATTTTGGATTATTTATA-3′; reverse: 5′-TACTAAATATCACCCCAAAA-3′. The PCR protocol included an initial denaturating cycle (3 min, 95 °C), followed by 30 cycles of denaturation (1 min, 95 °C), annealing (2 min, 51 °C), and extension (2 min, 72 °C), followed by a final extension cycle (10 min, 72 °C) terminating at 4 °C. Then, a second reaction was performed on the products of the first PCR primers using a second (nested) primers (forward: 5′-TTTTGGATTATTTATAGGATTTTGTTATAT-3′; reverse: 5′-CCCCAAAACTACTTCCTTACTTACA-3′) that bind to the target sequence and produce a 307-bp amplicon. The nested PCR product was subcloned using the TOPO TA cloning kit (Invitrogen) and transformed into *Escherichia coli* to produce 10 different colonies per plate. The presence of the DNA insert in the plasmid was determined with colony PCR, and plasmids containing the ligated *Gad1* promoter fragment were extracted with the QIAprep Spin Miniprep Kit (Qiagen). Sequence analysis by means of Sanger’s method was performed using the plasmids (10 plasmids per animal) at the Bioscience division, FASMAC Co, Japan.

### 4.7. Chromatin Immunoprecipitation Assay

Mice treated with either VPA or saline for 7 days were anesthetized deeply with isoflurane and the forebrain was rapidly dissected and stored at –80 °C on PND15. Approximately 25 mg of the forebrain tissue was minced on ice using a scalpel and incubated in 1.5% formaldehyde solution for 20 min at room temperature to cross-link the proteins to the DNA. The SimpleChIP Plus Enzymatic Chromatin IP Kit (#9005, Cell Signaling Technology, Tokyo, Japan) was used in the subsequent assay procedure. The cross-linked tissue was sonicated with a Bioruptor (UCD-250, Cosmo Bio, Tokyo, Japan) to reduce the size of DNA fragments to 150 to 900 bp and chromatin was immunoprecipitated using rabbit polyclonal antibody to MBD1 (1:50; sc-25261, Santa Cruz Biotechnology, Dallas, TX, USA), rabbit polyclonal antibody to MBD2 (1:50; ab38646, Abcam), or rabbit polyclonal antibody to MeCP2 (1:500; #07-013, Millipore). ChIP-Grade Protein G magnetic beads were used to enable the rapid isolation of protein/DNA complexes from the crude chromatin mixture. After reversal of cross-links with 5 M NaCl and proteinase K, 1/25 of DNA purified from ChIP was subjected to quantitative PCR (qPCR) using a primer set designed to target the mouse *Gad1* promoter region (forward: 5′-CACACACCCTCCTTTCTGGT-3′; reverse: 5′-GAAGGGAGAGATCCGGAGAG-3′).

### 4.8. Statistical Analysis

All statistical analyses were performed using SPSS Statistics version 22 on a PC. Simple comparisons between two groups were performed by independent Student’s *t*-test. Analysis of variance (ANOVA) was used for multiple comparisons among the 4 groups with respect to genotype, treatment, and genotype × treatment interaction. Bonferroni correction was performed to detect pairwise differences. Values are reported as means ± SE. The threshold for statistical significance was set at *p* < 0.05 for all analyses.

## Abbreviations

ANOVA	analysis of variance
AP	area postrema
cDNA	complementary DNA
DNMT	DNA methyltransferase
EGR1	Early growth response protein 1
GABA	γ-aminobutyric acid
GAPDH	glyceraldehyde 3-phosphate dehydrogenase
HDAC	histone deacetylase
MBD	Methyl-CpG binding domain protein
MeCP2	methyl-CpG-binding protein 2
NTS	nucleus tractus solitarius
PCR	polymerase chain reaction
RT-qPCR	quantitative reverse transcription PCR
RTT	Rett syndrome
RVLM	rostral ventrolateral medulla
Sp5	spinal trigeminal nucleus
TDG	thymine DNA glycosylase
TET	ten–eleven translocation
TSS	transcriptional start site
VPA	valproate
XII	hypoglossal nucleus

## Appendix A

**Table A1:** Respiratory parameters in WT and MeCP2^-/y^ mice injected with either saline or VPA for 7 days and measured at PND15.

	*n*	Breathing Frequency (Cycles/min)	**Tidal Volume (mL)**	Inspiratory Time (s)	Expiratory Time (s)	Peak Inspiratory Flow (mL/s)	Peak Expiratory Flow (mL/s)
WT—saline	6	244.6 ± 2.4	0.061 ± 0.001	0.073 ± 0.002	0.156 ± 0.002	1.165 ± 0.005	1.031 ± 0.020
WT—VPA	6	253.0 ± 4.7	0.051 ± 0.002	0.077 ± 0.002	0.167 ± 0.010	1.106 ± 0.024	1.036 ± 0.070
MeCP2^-/y^—saline	6	235.6 ± 3.4	0.063 ± 0.004	0.089 ± 0.002	0.184 ± 0.010	1.126 ± 0.041	1.076 ± 0.044
MeCP2^-/y^—VPA	6	239.0 ± 5.6	0.057 ± 0.001	0.085 ± 0.003	0.167 ± 0.004	1.062 ± 0.045	1.006 ± 0.028

The numbers of mothers that raised mice belonging to each group were 5 (WT-saline), 5 (WT-VPA), 6 (*Mecp2^-/y^*-saline), and 6 (*Mecp2^-/y^*-VPA), respectively. Littermates, if any, belonged to the same group. Values are means ± SE.

**Table A2:** Primer sequences used for RT-qPCR.

Gene		Sequences (F; Forward, R; Reverse)	Genbank Number
*Gad1*	F	5′-TTCTGGTACATTCCACAAAGCCTTC-3′	NM_008077.5
	R	5′-CCATGGTTGTTCCTGACTCCATC-3′	
*Tet1*	F	5′-TCAGCATGAAGTCTCAGTTGACTCC-3′	NM_001253857.2
	R	5′-GAATTGATGCCTTATTCCCACCA-3′	
*Tet2*	F	5′-GGTGCTACCCAGATTGCTCCA-3′	NM_001040400.2
	R	5′-TGGTCTAAGCCTCCACTGTTAGCTC-3′	
*Tet3*	F	5′-CTGTCCATCTCATGGAGCTTTC-3′	NM_001347313.1
	R	5′-GCGTATGCACCTCCAATGTGTTA-3′	
*Dnmt1*	F	5′-CTTCGGCAACATCCTGGACA-3′	NM_001199431.1
	R	5′-ACTGGACAGCAGGCAGAGCTTA-3′	
*Dnmt3a*	F	5′-GCATACAGCTTGCTGCACTCTC-3′	NM_153743.4
	R	5′-ACCTGCTGTACGCATTGACC-3′	
*Dnmt3b*	F	5′-TTGCTTTGGTACAAGGGCTGAA-3′	NM_001003960.4
	R	5′-TCATCCCTGCTGACATCATCATC-3′	
*Tdg*	F	5′-TAGGAAACGTGCGTGTTCAG-3′	NM_001358517.1
	R	5′-CTCATACTGCCAAACCAGCA-3′	
*Egr1*	F	5′-TCAGTGGCCACCACCTTTG-3′	NM_007913.5
	R	5′-AAAGGTCGCTGTCATGTCTGAA-3′	
*Gapdh*	F	5′-TGTGTCCGTCGTGGATCTGA-3′	NM_008084.3
	R	5′-TTGCTGTTGAAGTCGCAGGAC-3′	

**Table A3:** Statistical values concerning the expression of genes encoding enzymes that can modify DNA methylation and a transcription factor (EGR1).

	No. of Mice Belonging to Each Group				No. of Mothers that Raised Mice Belonging to Each Group				Results of A Two-Factor ANOVA		
Genes	WT-saline	WT-VPA	Mecp2^-/y^-saline	Mecp2^-/y^-VPA	WT-saline	WT-VPA	Mecp2^-/y^-saline	Mecp2^-/y^-VPA	geno-type	treat-ment	inter-action
*Tet1*	6	6	6	6	5	5	6	6	n.s.	*p* < 0.01	*p* < 0.05
*Tet2*	6	6	6	6	5	5	6	6	n.s.	n.s.	n.s.
*Tet3*	6	6	6	6	5	5	6	6	*p* < 0.01	n.s.	n.s.
*Dnmt1*	8	8	8	8	7	7	7	7	n.s.	n.s.	n.s.
*Dnmt3a*	11	12	10	12	10	10	10	11	n.s.	n.s.	n.s.
*Dnmt3b*	6	6	6	6	5	5	6	6	n.s.	n.s.	*p* < 0.01
*Tdg*	12	11	10	10	10	10	10	10	n.s.	n.s.	n.s.
*Egr1*	12	11	12	12	10	10	10	11	n.s.	n.s.	n.s.
